# Sjogren’s syndrome related autoimmune encephalopathy presenting as depression and involuntary agitation in a 15-year-old girl: a case report

**DOI:** 10.3389/fimmu.2023.1145948

**Published:** 2023-06-29

**Authors:** Yu-Tung Lee, Chao-Yi Wu, I-Jun Chou, Chun-Hao Liu

**Affiliations:** ^1^ Department of Psychiatry, New Taipei Municipal Tucheng Hospital, New Taipei City, Taiwan; ^2^ College of Medicine, Chang Gung University, Taoyuan City, Taiwan; ^3^ Division of Allergy, Asthma and Rheumatology, Department of Pediatrics, Chang Gung Memorial Hospital at Linkou, Taoyuan City, Taiwan; ^4^ Division of Pediatric Neurology, Chang Gung Children’s Hospital and Chang Gung Memorial Hospital, Chang Gung University College of Medicine at Linkou, Taoyuan City, Taiwan; ^5^ Department of Child and Adolescent Psychiatry, Chang Gung Memorial Hospital at Linkou, Taoyuan City, Taiwan

**Keywords:** adolescent, case report, depression, self-harm behaviors, Sjogren’s syndrome

## Abstract

**Introduction:**

Sjogren’s syndrome is an autoimmune disease that commonly involves exocrinopathy. Although studies have reported psychiatric manifestations resulting from Sjogren’s syndrome, few studies have focused on such manifestations in pediatric patients. Herein, we present a case of an adolescent girl with depression and involuntary self-harm behaviors related to Sjogren’s syndrome with central nervous system involvement.

**Case presentation:**

A 15-year-old girl, with an underlying history of epilepsy, developed depressive symptoms of a year’s duration, accompanied by three seizure episodes and involuntary self-harm behaviors. The self-harm behaviors, which included head banging and arm scratching, were sudden onset, involuntary, and unable to be recalled afterwards. After admission to our ward, the patient was positive for serum antinuclear antibodies and Schirmer’s test. Moreover, 24-hour electroencephalography revealed epileptiform discharges during the mood swing episodes. Positive findings for antinuclear antibodies and anti-SSA antibodies in both serum and cerebrospinal fluid, suggested central nervous system involvement in Sjogren’s syndrome. After rituximab treatment, her mood became euthymic, and her involuntary self-harm behaviors ceased.

**Conclusion:**

Central nervous system involvement leading to psychiatric presentations has rarely been reported in adolescents with Sjogren’s syndrome. When treating adolescent patients with involuntary self-harm behaviors and neurological symptoms, it is crucial to consider autoimmune encephalitis related to Sjogren’s syndrome in the differential diagnosis.

## 1 Introduction

Sjogren’s syndrome (SS) is a systemic autoimmune disease involving exocrinopathy. It is characterized by dry mouth, dry eyes, fatigue, and arthralgia. Other systemic manifestations such as skin rashes, peripheral neuropathy, and central nervous system (CNS) involvement occur in approximately 30% to 40% of patients with SS. On the basis of formal criteria, the estimated prevalence of SS in adults is 0.3 to 1 per 1000 persons worldwide (1), and 16.0 per 100000 persons in Taiwan (2). Epidemiologic data regarding pediatric-onset SS are limited; nevertheless, similar to adult patients, children with SS are predominately girls (3).

In addition to presenting with ocular and oral manifestations, a substantial proportion of patients with SS present with neuropsychiatric symptoms owing to CNS involvement (4, 5). In a comparative population-based study, up to 96% of patients with SS reported experiencing fatigue, depression, anxiety, and cognitive dysfunction (6). Psychiatric symptoms in children and adolescents with SS vary considerably. Hammett et al. reported four cases of adolescents with SS who had either psychosis or suicidal ideation (7), and Ong et al. published a case of an adolescent patient with obsessive compulsive disorder and major depressive disorder, which were associated with SS (8). Herein, we present a case of a patient with depression and involuntary agitation who was subsequently diagnosed as having SS; we also discuss the importance of timely diagnosis and management.

## 2 Case description

A 15-years-old girl with an underlying history of epilepsy was sent to our hospital because of intermittent mood dysregulation and agitation for 4 months. The patient had no family history of epilepsy or autoimmune disease. She experienced her first episode of generalized tonic-clonic seizure, which lasted for over 30 minutes, at the age of 8 years. She was diagnosed as having encephalopathy related to *Mycoplasma pneumoniae* infection. She then received intravenous methylprednisolone, followed by a short course of low dose oral prednisolone. No further seizure was noted after the treatment, and the patient was lost to follow-up with no further medical treatment. No personality change or cognitive dysfunction was noted in the intervening years.

One year before her current admission, the patient noticed a progressive decline in confidence, insomnia, and poor memory, leading to difficulties in her interpersonal relationships and academic performance. She suffered from low mood, general fatigue, poor concentration and several somatic complaints such as headaches and extremity weakness. She visited a psychiatric clinic and took bupropion 150mg, and aripiprazole 20mg/day. Three episodes of generalized tonic-clonic seizure occurred during her depressive periods, which prompted her to seek medical attention. She underwent electroencephalography (EEG) at our pediatric neurology clinic, and the EEG results revealed right centroparietal spikes. She was then administered a regimen of topiramate 200mg/day, and she claimed to have complete remission of her mood and could return to school after treatments.

Four months before the current admission, the patient complained of depressed mood, insomnia, poor appetite, poor concentration, and thoughts of helplessness. Moreover, she presented with sudden-onset agitation and self-harm behaviors, such as head banging or arm scratching, which she could not recall afterwards. The episodes occurred 3 times a day and lasted for 10 to 30 minutes each time. An aura involving machine sounds or flashes of light, occasionally preceded the mood and behavioral changes. She was admitted to our pediatric ward under the suspicion of seizure-induced behavioral change. Lamotrigine 10mg/day was added to her existing regimen of topiramate. A comprehensive neurological survey revealed no structural lesion on her brain magnetic resonance imaging scans and no abnormal findings in a cerebrospinal fluid (CSF) examination, but her 24-hour EEG revealed epileptiform discharges during the mood swings. Immunologic screening showed positive antinuclear antibodies (ANA, 1: 1280 speckled) and mildly decreased C3. Thyroid function, ovarian sonography, and tumor markers were within normal range. Surveys for viral infections were all negative. Under the impression of immune-mediated encephalopathy, methylprednisolone pulse therapy 1g/day was administered for 3 days, followed by oral prednisolone. However, her mood remission was only partial, and the involuntary agitation and self-harm behaviors occurred 2 months after discharge. She could not attend school owing to her depression and behaviors. When she later developed Steven-Johnson syndrome, lamotrigine was stopped and was replaced by valproic acid.

**Figure 1:**
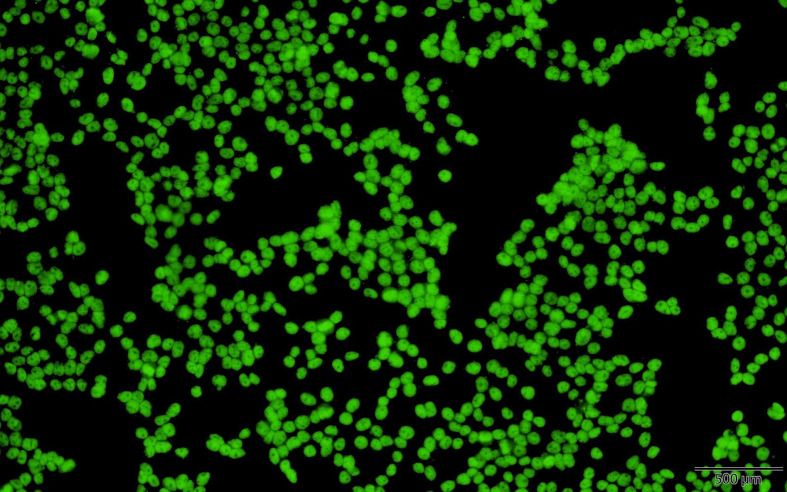
Positive finding for antinuclear antibodies in indirect immunofluorescence assay on HEp-2 cells.

Because of her aggravated clinical agitation and self-harm behaviors, the patient was again admitted to our pediatric ward. Prolonged QTc (479ms) was noted during evaluation for chest tightness. EEG revealed paroxysmal focal spikes over the right frontoparietal area. Her serum sample was positive for ANA with a titer of 1:1280, suggesting autoimmune encephalitis. She received topiramate 150mg/day and valproic acid 400mg/day for epilepsy control. She also received fluoxetine 10mg/day and aripiprazole 5mg/day for depression. Further survey for an autoimmune etiology revealed positive serum anti-SSA antibodies (67.2 U/mL), positive serum cryoglobulin IgG, IgM, and positive ANA in her CSF (**Figure 1**). Anti-Cardiolipin IgG, IgM, ribosome-P, anti-β2-glycoprotein I IgG, anti-RNP, anti-SMD, MPO, PR3, rheumatic factor, anti-double stranded DNA, C3, and C4 levels were within normal limits, and HBsAg, anti-HBs, anti-HBc, anti-TPO, anti-THYG, anti-TSHR, anti-HCV levels were all negative. Anti-neuronal autoantibodies including aquaporin-4 (AQP4) antibodies, myelin oligodendrocyte glycoprotein (MOG) antibodies, glutamate acid decarboxylase (GAD) antibody, anti-N-methyl-D-aspartate receptor (NMDAR) antibody, Voltage gated potassium channel (VGKC)-associated protein (CASPR2) antibodies, VGKC-associated protein (LGI1) antibodies, Glutamate receptors (type AMPA1 and AMPA2) antibodies, γ-aminobutyric acid type B (GABA_B_) receptor antibodies, Dipeptidyl aminopeptidase-like protein 6 (DPPX) antibodies were all negative. In accordance with her symptoms of dry eyes and dry mouth, the Schirmer’s test was positive (2mm). Single photon emission computed tomography (SPECT) revealed cortical hypometabolism at the bilateral prefrontal, bilateral parietal, and bilateral visual cortices. We assessed her CSF again for anti-SSA antibodies and had a positive finding (0.4 U/mL). The CSF/serum ratio of anti-SSA antibodies (0.006) was three times higher than that of IgG (2.88 mg/dl/1470 mg/dl = 0.002). This result suggested local production of antibodies in the CNS rather than only passive transmission of antibodies from serum (9). On the basis of patient’s clinical symptoms of ocular and oral dryness, positive findings for serum and CSF anti-SSA antibodies, and the Schirmer’s test results, SS was diagnosed in accordance with the 2017 ACR-EULAR classification criteria (1).

**Figure 2:**
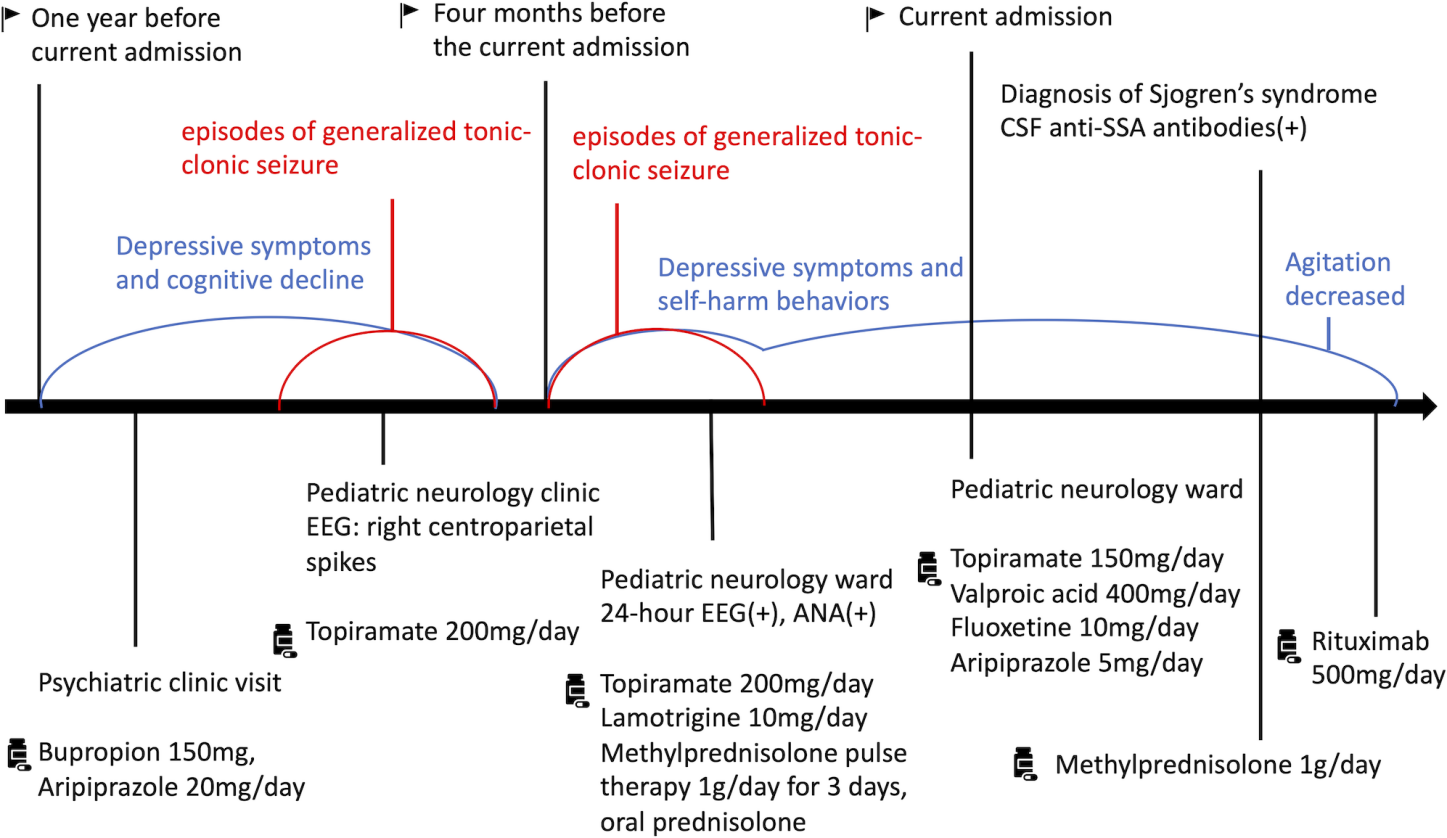
Specific historical events and information from this episode of care.

Under the impression of SS-related autoimmune encephalitis and QTc prolongation (10), intravenous methylprednisolone 1g/day was administered for 3 days. The frequency and intensity of agitation decreased from approximately eight times per day to twice a day. After the QTc returned to normal, rituximab 500mg was infused intravenously. She had no further agitation or self-harm behaviors after the treatment of methylprednisolone and rituximab. We kept her antidepressants and antipsychotics and discharged her. She did not exhibit any involuntary agitations during outpatient follow-up. The patient reported an improvement in mood and began to bring life back on track. **Figure 2** showed specific historical events and information in sequence.

## 3 Discussion

We report a case of an adolescent girl with SS who initially presented with worsening depressive symptoms, agitation, irritability, and involuntary self-harm behaviors. The presence of anti-SSA antibodies in CSF could be attributed to CNS involvement in SS. After immunosuppressant therapy with steroid and rituximab, her symptoms were relieved. Children and adolescents with autoimmune encephalitis commonly experience neuropsychiatric symptoms including behavioral, mood, or personality changes; sleep problems; cognitive dysfunction; and psychotic symptoms (11).

Studies have reported that up to 80% of patients with SS experience psychiatric symptoms or cognitive dysfunctions (12). Depression is common among adults with SS, with the estimated prevalence ranging between 8.33% and 75.56% (13). Andrianopoulou et al. found that disrupted global integrity and regional connectivity of multiple brain networks in the brain images could be related to depression, cognitive impairment, and attention and memory deficits among patients with SS (14). In our case, the SPECT image revealed cortical hypometabolism, which could be the evidence of brain involvement in adolescent with SS. Therefore, SS should be considered in adolescents experiencing depression and cognitive decline, especially when the symptoms are refractory to treatment and subsequent history, examination, and serology findings are consistent with SS (8).

Apart from gradual changes in mood and cognition, our patient exhibited self-harm behaviors during her uncontrollable agitation, which are difficult to recognize as a manifestation of SS at the beginning of the clinical course. Seizures are a common neurological presentation in children with autoimmune encephalitis (11). Although well-recognized, goal-directed ictal behavior is not common, Shih et al. described a rare case of a patient who presented with complex motor interaction and vocal features during a frontal lobe seizure (15). The patient in our case engaged in unconscious self-mutilation and experienced perceptual changes before the onset of agitation. She was amnestic for most of the events, although she could partially follow instructions. EEG during such episodes showed focal spikes over the right frontoparietal area, leading us to speculate that our patient’s uncontrollable agitation resulted from seizures associated with SS-related encephalitis and required a neurological survey for further evaluation.

Research has suggested that early intervention, such as the corticosteroids and immunosuppressive agents that we gave to our patient, could rapidly ameliorate symptoms and prevent permanent damage engendered by SS-related autoimmune encephalitis (11, 12). Moreover, rituximab is effective in depleting B cells, thus reducing autoantibodies such as the anti-SSA antibodies presenting in our patient (16).

Despite the evidence from CSF examination indicated the CNS involvement of SS, this paper has limitations. First, since her mood and cognitive symptoms came before her neurological symptoms, we could not confirm the patient’s autoimmune encephalitis caused by SS was the only contribution to her depressive symptoms and self-harm behaviors. Second, whether her self-harm behaviors were all involuntary needs more observation because the behaviors were complicated. However, the patient’s depressive symptoms and behaviors increased mostly with the start of neurological symptoms and decreased by seizure treatments or SS treatments. Therefore, the correlation between her clinical manifestation and SS diagnosis were strongly indicated.

## 4 Conclusion

Although several studies have already illustrated the correlation between SS and psychiatric symptoms, cases of adolescent patients with SS-related autoimmune encephalitis presenting as depression and involuntary self-harm behaviors are relatively rare. Autoimmune encephalitis—SS-related or otherwise—should be considered in patients with a refractory course of depression, memory deficits, and behavioral agitation along with neurological symptoms such as seizures. A detailed survey for various autoimmune diseases may facilitate early diagnosis and the provision of appropriate treatments to prevent further consequences.

## Data availability statement

The original contributions presented in the study are included in the article/supplementary material. Further inquiries can be directed to the corresponding authors.

## Ethics statement

The studies involving human participants were reviewed and approved by Chang Gung Medical Foundation Institutional Review Board. Written informed consent to participate in this study was provided by the participants’ legal guardian/next of kin. Written informed consent was obtained from the individual and her legal guardian for the publication of any potentially identifiable images or data included in this article.

## Author contributions

Y-TL, I-JC, and C-HL contributed to the conception and the design of this case report, and Y-TL wrote the first draft of the manuscript. C-YW, I-JC, and C-HL substantively revised the manuscript and added relevant data and references. All authors contributed to the article and approved the submitted version.

## References

[1] Mariette X Criswell LA . Primary sjögren's syndrome. N Engl J Med (2018) 378(10):931–9. doi: 10.1056/NEJMcp1702514 29514034

[2] Yu KH See LC Kuo CF Chou IJ Chou MJ . Prevalence and incidence in patients with autoimmune rheumatic diseases: a nationwide population-based study in Taiwan. Arthritis Care Res (2013) 65(2):244–50. doi: 10.1002/acr.21820 22899470

[3] Tucker L Ciurtin C . Sjögren syndrome and immunoglobulin-G4 disease. In: Textbook of pediatric rheumatology. Philadelphia, PA: Elsevier (2021).

[4] Delalande S de Seze J Fauchais AL Hachulla E Stojkovic T Ferriby D . Neurologic manifestations in primary sjögren syndrome: a study of 82 patients. Medicine. (2004) 83(5):280–91. doi: 10.1097/01.md.0000141099.53742.16 15342972

[5] Devinsky O Schein A Najjar S . Epilepsy associated with systemic autoimmune disorders. Epilepsy currents (2013) 13(2):62–8. doi: 10.5698/1535-7597-13.2.62 PMC3639560 23646005

[6] Harboe E Tjensvoll AB Maroni S Gøransson LG Greve OJ Beyer MK . Neuropsychiatric syndromes in patients with systemic lupus erythematosus and primary sjögren syndrome: a comparative population-based study. Ann rheumatic diseases (2009) 68(10):1541–6. doi: 10.1136/ard.2008.098301 18930990

[7] Hammett EK Fernandez-Carbonell C Crayne C Boneparth A Cron RQ Radhakrishna SM . Adolescent sjogren’s syndrome presenting as psychosis: a case series. Pediatr Rheumatol (2020) 18(1):15. doi: 10.1186/s12969-020-0412-8 PMC7014743 32046763

[8] Ong LTC Galambos G Brown DA . Primary sjogren's syndrome associated with treatment-resistant obsessive-compulsive disorder. Front Psychiatry (2017) 8:124. doi: 10.3389/fpsyt.2017.00124 28744230 PMC5504162

[9] Kurotaki K Fujita M Aizawa T Tsugawa K Tanaka H . Aseptic meningitis as initial presentation of subclinical sjögren's syndrome: could the cerebrospinal fluid anti-Ro/SSA and anti-La/SSB antibody system be the culprit? Modern Rheumatol Case Rep (2022) 6(2):217–9. doi: 10.1093/mrcr/rxac011 35134231

[10] Lazzerini PE Cevenini G Qu YS Fabris F El-Sherif N Acampa M . Risk of QTc interval prolongation associated with circulating anti-Ro/SSA antibodies among US veterans: an observational cohort study. J Am Heart Assoc (2021) 10(4):e018735. doi: 10.1161/JAHA.120.018735 33533258 PMC7955337

[11] Rosello R Girela-Serrano B Gómez S Baig B Lim M Taylor S . Characterizing the features and course of psychiatric symptoms in children and adolescents with autoimmune encephalitis. Eur Arch Psychiatry Clin Neurosci (2022) 272(3):477–82. doi: 10.1007/s00406-021-01293-5 PMC8938365 34272976

[12] Cox PD Hales RE . CNS sjögren's syndrome: an underrecognized and underappreciated neuropsychiatric disorder. J neuropsychiatry Clin neurosciences (1999) 11(2):241–7. doi: 10.1176/jnp.11.2.241 10333995

[13] Cui Y Li L Yin R Zhao Q Chen S Zhang Q . Depression in primary sjögren's syndrome: a systematic review and meta-analysis. Psychology Health Med (2018) 23(2):198–209. doi: 10.1080/13548506.2017.1339895 28621153

[14] Andrianopoulou A Zikou AK Astrakas LG Gerolymatou N Xydis V Voulgari P . Functional connectivity and microstructural changes of the brain in primary sjögren syndrome: the relationship with depression. Acta radiologica (Stockholm Sweden 1987) (2020) 61(12):1684–94. doi: 10.1177/0284185120909982 32212831

[15] Shih JJ LeslieMazwi T Falcao G Van Gerpen J . Directed aggressive behavior in frontal lobe epilepsy: a video-EEG and ictal spect case study. Neurology. (2009) 73(21):1804–6. doi: 10.1212/WNL.0b013e3181c2933f PMC2849702 19846832

[16] Meijer JM Meiners PM Vissink A Spijkervet FK Abdulahad W Kamminga N . Effectiveness of rituximab treatment in primary sjögren's syndrome: a randomized, double-blind, placebo-controlled trial. Arthritis rheumatism (2010) 62(4):960–8. doi: 10.1002/art.27314 20131246

